# Minimum Information about a Genotyping Experiment (MIGEN)

**DOI:** 10.4056/sigs.1994602

**Published:** 2011-11-22

**Authors:** Jie Huang, Daniel Mirel, Elizabeth Pugh, Chao Xing, Peter N. Robinson, Alexander Pertsemlidis, LiangHao Ding, Julia Kozlitina, Joseph Maher, Jonathan Rios, Michael Story, Nishanth Marthandan, Richard H. Scheuermann

**Affiliations:** 1 Pathology, University of Texas Southwestern Medical Center, Dallas, TX, USA; 2 McDermott Center for Human Growth and Development, University of Texas Southwestern Medical Center, Dallas, TX, USA; 3 Radiation Oncology, University of Texas Southwestern Medical Center, Dallas, TX, USA; 4 Internal Medicine, University of Texas Southwestern Medical Center, Dallas, TX, USA; 5 Clinical Science, University of Texas Southwestern Medical Center, Dallas, TX, USA; 6 The Broad Institute Center for Genotyping and Analysis, Broad Institute of MIT & Harvard, Cambridge, MA, USA; 7 Institute of Genetic Medicine, Johns Hopkins School of Medicine, Baltimore, MA, USA; 8 Institut für Medizinische Genetik, Charité - Universitätsmedizin Berlin, Berlin, Germany

## Abstract

Genotyping experiments are widely used in clinical and basic research laboratories to identify associations between genetic variations and normal/abnormal phenotypes. Genotyping assay techniques vary from single genomic regions that are interrogated using PCR reactions to high throughput assays examining genome-wide sequence and structural variation. The resulting genotype data may include millions of markers of thousands of individuals, requiring various statistical, modeling or other data analysis methodologies to interpret the results. To date, there are no standards for reporting genotyping experiments. Here we present the Minimum Information about a Genotyping Experiment (MIGen) standard, defining the minimum information required for reporting genotyping experiments. MIGen standard covers experimental design, subject description, genotyping procedure, quality control and data analysis. MIGen is a registered project under MIBBI (Minimum Information for Biological and Biomedical Investigations) and is being developed by an interdisciplinary group of experts in basic biomedical science, clinical science, biostatistics and bioinformatics. To accommodate the wide variety of techniques and methodologies applied in current and future genotyping experiment, MIGen leverages foundational concepts from the Ontology for Biomedical Investigations (OBI) for the description of the various types of planned processes and implements a hierarchical document structure. The adoption of MIGen by the research community will facilitate consistent genotyping data interpretation and independent data validation. MIGen can also serve as a framework for the development of data models for capturing and storing genotyping results and experiment metadata in a structured way, to facilitate the exchange of metadata.

## Introduction

With the continued advances in genotyping technologies, especially high-throughput genotyping techniques, there is an exponential growth of genetic data in the biomedical literature [1]. This information forms a foundation for biomedical researchers to formulate new hypotheses about the molecular determinants of disease pathogenesis. However, the diversity between genotyping experiments, including experimental design, assay technique and data analysis method, makes data interpretation, validation and reproduction difficult to the end-users. The information reported in the literature for genotyping experiments is often insufficient, ambiguous and inconsistent. The research community lacks genotyping experiment reporting standards, so called minimum information checklist.

A minimum information checklist specifies the minimum set of information required to describe experimental findings, such that reviewers and end-users of the scientific publication can interpret and use the experimental results unambiguously. The research community is increasingly in favor of application of minimum information checklists [2]. Successful implementation and adoption of minimum information checklists by journals and databases include the Minimum Information About a Microarray Experiment (MIAME) [3] and the Minimum Information about a Flow Cytometry Experiment (MIFlowCyt) [4] standards. Currently, there is no equivalent checklist for reporting genotyping experiments. To establish such standard, here we propose the Minimum Information about a Genotyping Experiment (MIGen). MIGen is developed to specify a set of minimum information that need to be provided by the author of a genotyping experiment, either when publishing in a journal article or when making data available in public databases. MIGen is a registered project under Minimum Information for Biological and Biomedical Investigations (MIBBI [5]), which coordinates the development of minimum information checklists for biological and biomedical research.

MIGen is developed by cross-disciplinary experts in clinical and basic biological research, bioinformatics and biostatistics. It is proposed to the research community to collect comments and to reach consensus.

## Challenge of MIGen Development

In MIGen, a genotyping experiment is defined as a study that is designed to elucidate some aspect of the genomic nucleotide sequence structure of an individual or group of individual organism(s). Genotyping experiments covered by MIGen are highly diverse in many aspects:

- Genotyping experiment techniques are employed to accomplish different study purposes. They may be used as a primary discovery method, as in a genome-wide association study (GWAS), or to test the association or effect of specific sequence variants known to contribute to a phenotype of interest, as in studies utilizing animal model of disease.
- Different types of genetic variants may be assayed in genotyping experiments, including single nucleotide polymorphisms (SNP), variable numbers of tandem repeats (e.g. microsatellites), copy number variation (CNV), genomic rearrangements, transgenes, gene knockout, etc.. Genotyping scale range from a small number of genomic variants genotyped in only a few biological samples to millions of variants assayed in thousands of samples.
- Depending on the purpose of a genotyping study, the experimental design varies - population study versus familial study, prospective study versus retrospective study, etc. - requiring different subject selection criteria and different subject/population characteristics captured during the course of the study.
- Genotyping assay techniques also differ substantially. They differ in their technical complexity and in the type of raw data generated. Assay techniques range from single PCR amplification assays to various high throughput approaches. The type of raw data generated also varies, for example, from Sanger sequencing technique which generates one chromatogram read per sample, to next generation sequencing methods which generate large numbers of short reads, provided as fluorescent image files.
- Multiple data processing and analysis methods exist to accommodate the diversity between genotyping experiments. The data analysis procedures of a genotyping experiment may range from a simple call from a PCR amplicon size to a complex sequence of steps that may include quality control filtering, imputation, population stratification and statistical test.

A minimum information checklist should be short and complete, so that it is convenient for users to follow, yet specifies all of the elements required to describe an experiment of the type. Given the diversity of genotyping experiments, as mentioned above, it is a significant challenge to develop MIGen as a concise but generalized guideline to suit all kinds of genotyping experiment reports. Moreover, genotyping technologies and their applications evolve rapidly, so MIGen should also be flexible to accommodate future genotyping technologies.

## MIGen Development Principles

### Components of MIGen

MIGen was designed to be consistent with other well-established MIBBI projects, e.g. MIAME, MIFlowCyt and MISFISHIE [6].

MIGen consists of four sections:
  1. **Experiment Overview** specifies general information that should be provided for the overall experiment, e.g., experiment purpose, study personnel, study centers, etc.
  2. **Experiment Subjects Description** specifies information that should be provided to unambiguously interpret how the experimental subjects were recruited and selected, as well as subject and population characteristics collected during the study.
  3. **Genotyping Procedure** provides guidelines to report how biological samples were collected and processed from experimental subjects and how the raw data was generated. This section includes descriptions of the genomic variants assayed and descriptions of genotyping procedures and technologies.
  4. **Data Transformation** section includes specification for reporting the data processing and analysis methods.

The first version of MIGen can be found on the MIGen website [7]. Like other MIBBI standards, we emphasize that MIGen specifies the minimum information that needs to be reported, but not the order or format of how the required information is provided. Therefore, when using the MIGen standard to guide the reporting of genotyping experiments, it is not necessary to organize the report following MIGen document structure. Moreover, MIGen states that one shall refer to the appropriate minimum information document if one exists for a specific experiment technique involved in the genotyping procedure, e.g., refer to the MIFlowCyt standard if a flow cytometry technique was used; refer to the MIAME standard if a microarray technique was used.

### Ontology for Biomedical Investigation application in MIGen

**Figure 1:**
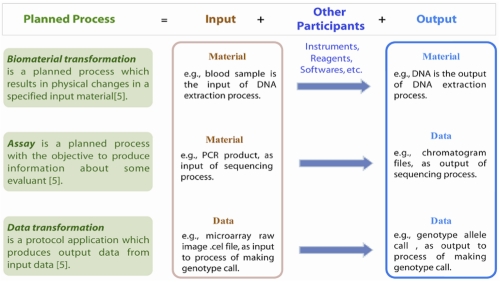
Planned Processes

Of the four sections covered in MIGen, the first two sections are essentially applicable to all types of genotyping experiments and were relatively straightforward to develop. In contrast, because of the complex nature of genotyping assay techniques and various data analysis methods employed, the Genotyping Procedure and Data Transformation sections are the major challenge in MIGen development. To capture the common features of the Genotyping Procedure and Data Transformation sections for all genotyping experiments, MIGen applies the “planned process” concept from the Ontology for Biomedical Investigations (OBI) [8]. A planned process is a processual entity that realizes a plan, which is the concretization of a plan specification (ID: “obo:OBI_0000011”) [8]. There are three basic types of planned processes in OBI: biomaterial transformation, assay and data transformation, each of which is a process with three components: input, other participants and output, as illustrated in Figure 1.

The biomaterial transformation process is defined as an event with one or more biomaterials as inputs and outputs. For example, DNA extraction from a blood sample is a biomaterial transformation process, where blood is the input biological material, DNA is the output material and the DNA extraction reagents and devices used in the process are other participants. An assay is a planned process with the objective to produce information about some evaluant (ID: “obo:OBI_0000070”) [8]. It has biological material as input and data as output. For example, a microarray based genotyping assay has DNA as input and raw image data as output, where reagents, instruments and software utilized in the process are other participants. Starting with the raw data generated from the assay, we move to the data transformation processes. A data transformation process is a protocol application that produces output data from input data (ID: “obo:OBI_0200000”) [8].

With the application of OBI concepts in MIGen, genotyping procedure and data analysis components of a genotyping experiment are considered as a sequence of planned processes, each of which can be categorized as a biomaterial transformation, assay, or data transformation process. With this abstractive view, virtually all steps executed in any genotyping experiment can be easily and explicitly specified at a high level, describing what information is required to be reported, without enumerating all the varieties for any given step. For example, MIGen specifies that if the input is a biomaterial, one must provide information on its type, its amount in value-unit pair, and other significant attributes. For detailed specification, please refer to the MIGen documentation.

The application of the OBI ontology within MIGen provides an abstractive framework that is generalized to define the necessary reporting standards for any process in a genotyping experiment. However, due to the complexity of genotyping experiments, there are many steps or processes involved, which can be reported at different levels of granularity depending on the experimenter’s definition of a process. For example, a PCR genotyping experiment can be broken down into sequential processes as following, starting from DNA sample: 1) *biomaterial transformation* where DNA is the input and assembled PCR reaction mix is the output, 2) *biomaterial transformation* where DNA sample in the PCR reaction mix is the input, the amplified DNA amplicon is the output of the thermocycler reaction, 3) an *assay* process where DNA amplicons are the input and the gel image is the output of the electrophoresis assay and 4) a *data transformation process* where the gel image was analyzed to determine the size of the samples’ amplicon. Alternatively, an experimenter can define the entire chain of processes as a single *assay* process where DNA (biological material) is the input and the size of each samples’ amplicon (data) is the output.

MIGen does not constrain how the experimenters break down the experimental or data analysis procedures, but rather specifies the list of key information that needs to be included to ensure the unambiguous interpretation, reproduction and reuse of the data.

## Hierarchical Structure of MIGen

**Figure 2:**
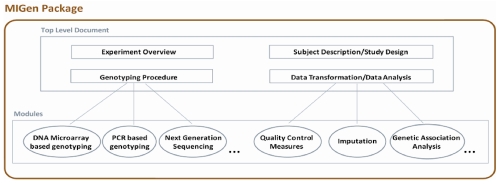
Hierarchical structure of MIGen package

Unlike many existing minimum information checklists for other experimental domains, such as MIAME for microarray gene expression experiments and MIFlowCyt for flow cytometry experiments, where the experimental techniques and raw data are relatively comparable, the huge diversity of genotyping experiments makes it difficult to list all of the key information of each genotyping experiment, especially for the Genotyping Procedure section. The introduction of OBI concepts into MIGen development solves the problem of how to provide a generalized guideline for all types of genotyping experiments, although at the same time it implies that the experimenters need to make judgments on what key information need to be reported for each experiment. To make certain that MIGen serves well as a minimum information checklist to ensure key information availability for each type of genotyping experiment technique and data analysis procedure, MIGen is built as a hierarchically structured package, as illustrated in Figure 2.

The top-level components in the hierarchy are the most general specification and are applicable to all different types of genotyping experiments, and are where OBI concepts are introduced. The next level components, which we call modules, inherit guidelines from the top-level specification with added concrete details of the particular experimental technique/analysis methods. This modular approach is mainly applied to the Genotype Procedure and Data Transformation sections of MIGen. With the hierarchical structure of MIGen, new modules can be added at anytime as the need arises for the research community.

The top-level hierarchical components of MIGen are available to the research community at [7]. The development of each specific module is ongoing among the authors.

## Discussion

The efficient sharing and exchange of data rely on consistent data reporting standards, with which end-users of the data can acquire an understanding of the data generation and analysis methods. With today’s high throughput genotyping technologies and large-scale genomic studies, data reusability is critical for cost effective study design and meta-analyses of published genetic studies. The development and implementation of MIGen standards fills the gap between the initial genotyping experiment information providers and the genotyping data users.

While MIGen follows the high-level structure of other well-established minimum information checklists, it also leverages foundational concepts from the OBI ontology. The use of planned processes and a hierarchical structure allows MIGen to accommodate the many varied and unique aspects of different genotyping experiments. A similar but distinct hierarchical architecture of checklists has been proposed by the Geomic Standards Consortium community, where the minimum information about any (x) sequence (MIxS) was created by reverse engineering an “overarching framework” [9] to serve as a single entry point for different technology-specific checklists, such as Minimum information about a marker gene sequence (MIMARKS) [9], the minimum information about a genome sequence (MIGS) [10], etc. Independently developed checklists are collected under the MIxS, sharing the same central set of core descriptors but having checklist specific descriptors as well. The MIGen hierarchical architecture not only provides a means for all modules to share common high-level structure, but also the specifications provide the guidelines for development of each module.

Further discussion within the research community must take place to reach the final consensus on the proposed standard. We welcome comments on the documentation and additions to the MIGen modules for specific genotyping experiment types. MIGen will facilitate data sharing in the research community, making independent data interpretation, validation and reproduction more efficient and unambiguous. MIGen can also serve as a framework for the development of data models to capture and store genotyping result data and experiment metadata in a structured way, to facilitate data exchange and sharing.
